# Dataset on factors associated with social cohesion of urban life in Jakarta

**DOI:** 10.1016/j.dib.2023.109339

**Published:** 2023-06-22

**Authors:** Sulfikar Amir, Irma Hidayana, Zahira Rahvenia, Salman Haydar

**Affiliations:** a School of Social Sciences, Nanyang Technological University, 48 Nanyang Avenue SHHK-05-31, Singapore 639818; b Department of Asian Studies/Public Health, St. Lawrence University, Canton, New York, United States of America; c LaporCovid19.org- Bona Indah Plaza A2-B11, Lebak Bulus, Jakarta, Indonesia; d Division of Communication, Informatics, and Statistics (Diskominfotik), the Provincial Government of Jakarta, Jl. Merdeka Selatan kav 8-9, Gedung Balaikota Blok G Lt. 13, Jakarta, Indonesia

**Keywords:** Social cohesion, Urban community, Trust, Recognition, Neighborhood, Jakarta

## Abstract

The data article examines the level of social cohesion among neighborhood communities in the urban context. Increased socioeconomic challenges of urban life affect how urban citizens interact one another, shaping their daily behavior as a cohesive urban society. Social cohesion is measured against five variables, namely trust, recognition, participation, reciprocity, and insertion. This dataset provides a closer look into how citizens of the megacity of Jakarta, Indonesia build relationships at the individual level to negotiate their social and cultural differences. It presents the pattern of social cohesion that binds the entire Jakarta population. The present dataset includes two thousand and fifty-two (2,052) survey questionnaires from Jakarta citizens. The data was collected proportionately from forty-four (44) districts (kecamatan) of Jakarta by using stratified random sampling techniques. This article includes information on reliability and factor loadings, as well as results of regression analyses.

**Specifications Table**

**Table:** 

Subject	Social Sciences
Specific subject area	Sociology
Type of data	Tables and Figures
How the data were acquired	Survey Questionnaire (questionnaire included in NTU Data Repository)
Data format	Raw, analyzed
Description of data collection	The data were collected from 23 January to 14 February 2022 in Jakarta, Indonesia using stratified random sampling. A survey questionnaire was shared in-person by enumerators with selected residents at all Sub-District Offices (Kantor Kelurahan) across the city ofJakarta. Each respondent filled out the questionnaire on site using a digital device provided by the assigned enumerator. The survey generated 2,052 responses.
Data source location	• Institution: Division of Communication, Information, and Statistics (Diskominfotik), the Provincial Government of Jakarta • City/Town/Region: Jakarta • Country: Indonesia. • Latitude and longitude for collected samples/data: -6.180692680377055, 106.8283066269856
Data accessibility	Repository name: DR-NTU (Data) Data identification number: DOI: https://doi.org/10.21979/N9/6FRF3J

## Value of the Data

• The survey dataset can be used to examine how the cohesiveness of citizens living in a megacity is built through daily interactions across neighborhoods and communities by looking into five key variables of social cohesion, namely trust, recognition, participation, reciprocity, and insertion.
• The survey dataset is valuable for city governments, policymakers, and urban researchers who are interested in understanding the pattern of social cohesion shaped by the socioeconomic and spatial conditions of urban life.
• The survey dataset is useful for designing urban programs to improve social cohesion between different groups living in the megacity.
• The survey dataset provides key indicators of social cohesion that can be used for urban planning and design aimed at strengthening cohesive interactions between citizens.

## 1 Objective

The objective of this dataset is to provide researchers with high-quality data on social cohesion of urban life in a megacity at the individual level. Social cohesion is measured on five variables, namely trust, recognition, participation, reciprocity, and insertion. This is the first large-scale survey conducted in Jakarta, Indonesia that contains representative data across districts. The data aims to show the patterns of cohesivity as a result of daily interactions between individual citizens across socioeconomic groups.

## 2 Data Description

### 2.1 Data

The dataset can provide insight into factors associated with social cohesion. Using regression analyses, structural equation modeling and factor analysis were applied to validate the construct and the relations between social cohesion and demography data. Tables 1 through 6 show demographic statistics, correlation coefficients, factor loadings, construct validity construct, discriminant validity, and Hetero Trait and Mono Trait (HTMT) analyses, respectively.

**Table 1:** Demographics table.

		Frequency	Percent	Total (%)
Gender	Female	1032	50.3	50.3
	Male	1020	49.7	100.0

Age	18-19	40	1.9	1.9
	20-24	139	6.8	8.7
	25-29	208	10.1	18.9
	30-34	239	11.6	30.5
	35-39	257	12.5	43.0
	40-44	359	17.5	60.5
	45-49	316	15.4	75.9
	50-54	260	12.7	88.6
	55-59	120	5.8	94.4
	60-64	66	3.2	97.7
	65+	48	2.3	100.0

Education	Not finished any school	5	0.2	0.2
	Not finished elementary school	23	1.1	1.4
	Elementary School	131	6.4	7.7
	Junior High School	266	13.0	20.7
	Senior High School	1179	57.5	78.2
	Diploma I/II	40	1.9	80.1
	Associate Degree/Diploma III	110	5.4	85.5
	Diploma IV/Undergraduate	282	13.7	99.2
	Master/Postgraduate	16	0.8	100.0

Monthly Income (Indonesian Rupiah)	< Rp 1.500.000	533	26.0	26.0
	Rp 1.500.001 - Rp 4.000.000	602	29.3	55.3
	Rp 4.000.001 - Rp 6.500.000	729	35.5	90.8
	Rp 6.500.001 - Rp 9.000.000	108	5.3	96.1
	Rp 9.000.001 - Rp 14.000.000	42	2.0	98.1
	Rp 14.000.001 - Rp 20.000.000	20	1.0	99.1
	Rp 20.000.001 - Rp 25.000.000	10	0.5	99.6
	> Rp 25.000.001	8	0.4	100.0

Was born in Jakarta	Yes	1442	70.3	70.3
	No	610	29.7	100.0

Was raised in Jakarta	Yes	1781	86.8	86.8
	No	271	13.2	100.0

Religion	Muslim	1940	94.5	94.5
	Christian Protestant	76	3.7	98.2
	Catholic	14	0.7	98.9
	Christian	1	0.0	99.0
	Buddhist	12	0.6	99.6
	Konghucu	1	0.0	99.6
	Don't have religion	1	0.0	99.7
	Do not answer	7	0.3	100.0

Residence Location	Main road	51	2.5	2.5
	Roadway (can be passed by two-way cars)	321	15.6	18.1
	Small road (only one way for car)	645	31.4	49.6
	Alley (only one way for motorbikes)	998	48.6	98.2
	Alleyway (just for walking)	37	1.8	100.0

*Note:* The eight (8) demographic variables were coded in data as Gender (1-Female, 2-Male) Age (1-15-19, 2-20-24, 3-25-29, 4-30-34, 5-35-39, 6-40-44, 7-45-49, 8-50-54, 9-55-59, 10-60-64, and 11>65), Education (1-not finished any school, 2-not finished Elementary School, 3-Elementary School, 4-Junior High School, 5-Senior High School, 6-Diploma I/II, 6-Associate Degree/Diploma III, 7-Diploma IV/Undergraduate, 8-Master's/postgraduate), monthly income in Indonesian rupiah currency (1-IDR< 1.500.000, 2-IDR 1.500.001 - Rp 4.000.000, 3-IDR 4.000.001 - IDR 6.500.000, 4-IDR 6.500.001 - IDR 9.000.000, 5-IDR 9.000.001 - IDR 14.000.000, 7-IDR 14.000.001 - IDR 20.000.000, 8-UDR 20.000.001 - IDR 25.000.000, and 9- >IDR 25.000.001), Born in Jakarta (1-Yes, 2-No), Raised in Jakarta (1-Yes, 2-No), Religion (1-Islam, 2-Christian Protestan, 3-Catholic, 4-Christian, 5-Buddhist, 6-Konghucu, 7-Don't have religion, 8-do not answer), and Residence location (1-main road, 2-roadway (can be passed by two-way cars), 3-small road (only one way for car), 4-Alley (only one way for bikes), 5-Alleyway (just for walking)

### 2.2 Demographic

Table 1 shows demographic statistics for the 2,052 respondents. The gender breakdown of female and male respondents was approximately 50.3% females and 49.7% males. Most respondents graduated from senior high school at their highest education level (57.5%), were between the ages of 40 and 44 (17.5%), and earned between IDR 4.000.001 and IDR 6.500.000 (35.5%) each month. Seventy-point-three percent of the participants were born in Jakarta, and 87.8 % were raised in Jakarta. Participants indicated their religion as Muslim (94.5%), Christian protestant (3.7%), Catholic (0.7%), Buddhist (0.6%), and 0.3% did not indicate their religions. Regarding residence location, 48.6% of participants lived in alley areas, 31.4% in small road areas, 15.6% in roadway areas, 2.5% in main road areas, and 1.8% in alleyway areas.

Table 2 provides information on the validity of the variables and factor loadings (factor correlation coefficients). Each social cohesion variable consists of two sub-variables. The five variables include trust, recognition, participation, reciprocity, and insertion. The two sub-variables trusts consist of trusting in leaving the house to neighbors (Tr_1) and the number of the family known (Tr_2); the recognition sub-variables include getting along with neighbors (Rc_1) and actively participating in the neighborhood (Rc_2); the two sub-variables of reciprocity consist of helping neighbors (Rp_1) and being helped by neighbors (Rp_2); the two sub-variables of participation include making donations to the community (Pt_1) and responsible to the community (Pt_2); and the two sub-variables of insertion consist of convenient living with the community (In_1) and feeling welcome by the community (In_2). The majority factor loads on the social cohesion variable are greater than .50, and an alpha coefficient greater than .70 suggests internal consistency. The factor load on the Trust variables was 0.3, and 0.4 yielded no alpha coefficients, suggesting lower internal consistency. Overall, KMO and Bartlett's Test value also suggest a substantial correlation in the data.

**Table 2:** Factor loading and validity.

Variables	Code	Factor Loading	ᵅ	CR	AVE
Trust	Tr_1	0.354		0.295	0.176
	Tr_2	0.476			

Recognition	Rc_1	0.583	0.673	0.700	0.548
	Rc_2	0.870			

Participation	Pr_1	0.695	0.719	0.722	0.566
	Pr_2	0.806			

Reciprocity	Rp_1	0.858	0.722	0.735	0.585
	Rp_2	0.659			

Insertion	In_1	0.893	0.906	0.907	0.829
	In_2	0.928			

Kaiser-Meyer-Olkin Measure of Sampling Adequacy					0.772

Bartlett's Test of Sphericity			Approx. Chi-Square		9621.551
			df		153
			Sig.		0

Table 3 presents evidence for discriminant validity. All values except the Insertion variable are less than .85, this suggests discriminant validity exists between SC constructs.

**Table 3:** Discriminant validity.

		1	2	3	4	5	6
1	Trust	0.419					
2	Recognition	0.368	0.741				
3	Participation	0.256	0.598	0.753			
4	Reciprocity	0.355	0.443	0.451	0.765		
5	Insertion	0.392	0.372	0.359	0.400	0.911	
6	Demographic	0.170	0.184	0.202	0.088	0.115	0.337

*Note:* Latent variable “demographics” consist of 10 variables i.e. Gender, Age, Education, Monthly income, was born in Jakarta, Was raised in Jakarta, Religion, and Residence location as detailed in Table 1.

Table 4 shows the results of HTMT analyses, which help establish discriminant validity.

**Table 4:** HTMT Ratio.

		1	2	3	4	5	6
1	Trust						
2	Recognition	0.852					
3	Participation	0.566	0.836				
4	Reciprocity	0.781	0.647	0.639			
5	Insertion	0.768	0.474	0.446	0.499		
6	Demographic	0.261	0.249	0.259	0.076	0.049	

*Note:* Latent variable “demographics” consist of 10 variables i.e. Gender, Age, Education, Monthly income, was born in Jakarta, Was raised in Jakarta, Religion, and Residence location as detailed in Table 1.

## 3 Experimental Design, Materials and Methods

The Provincial Government of Jakarta's Unit of Statistics facilitated the recruitment of participants. All participants signed a digital informed consent form before in-person survey were done to collect the data from 23 January to 14 February of 2022. Stratified random sampling was applied to select 2,052 respondents out of 8,4 million population in 44 districts (Kecamatan) of five municipalities across the capital city of Jakarta (West Jakarta, South Jakarta, East Jakarta, North Jakarta, and Central Jakarta). Respondents were required to answer a total of 21 survey items; thus no missing data was reported. Demographic and social cohesion variables data were gathered from the respondents. The survey instrument appears in Supplementary Material.

Enumerators of the Jakarta Provincial Government's Unit of Statistics visited respondents, and asked questions according to the questionnaire. Participants’ demographic characteristics were assessed through eight socio-demographic questions. Further, the social cohesion construct which includes trust, recognition, participation, reciprocity, and insertion variables were assessed through 10 set of questions. The questionnaires were adopted from the extant literature [1], [2], [3] and can be found in the supplementary material. The social cohesion framework was adopted from Chuang et al. [4] and Paramita et al. [5]. It has been constructed to be suitable for the purpose of the present study.

The trust variable consists of two sub-variables: “trusting in leaving the house to neighbors (Tr_1)” and “the number of the family well known (Tr_2)”. The former was measured on a 5-point scale ranging from (1) strongly distrust to (5) strongly trust, the latter ranging from (1) none to (5) over 10 families. The recognition's both sub-variables (“getting along with neighbors (Rc_1)” and “actively participating in the neighborhood (Rc_2)”) and the reciprocity's variables of “help neighbors (Rp_1)” and “helped by neighbors (Rp_2)” were measured on a 5-point scale ranging from (1) never to (5) very frequently. One of the participation's sub-variables, “making donations to the community (Pt_1)” was measured on a 5-point scale ranging from (1) never to (5) very frequently, while the other sub-variable, “responsible to the community (Pt_2)” was measured on a 5-point scale ranging from (1) completely doesn't care to (5) strongly care. In addition, both sub-variables of insertion, “convenient living with the community (In_1)” and “feel welcome by the community (In_2)” were measured on a 5-point scale ranging from (1) very uncomfortable to strongly comfortable. SPSS (v.26) were used to generate descriptive statistics, correlation in Table 6, regression in Table 5, reliability, discriminant validity, and HTMT ratio.

**Table 5:** Regresion.

					CI	
	Coefficients	Std. Error	t	Sig	Lower	Uper
SC <- D1	-0.002	0.040	0.071	0.944	-0.082	0.076
SC <- D2	-0.04	0.011	1.746	0.081	-0.041	0.002
SC <- D3	0.051	0.009	2.259	0.024	0.003	0.037
SC <- D4	-0.068	0.03	2.752	0.006	-0.142	-0.024
SC <- D5	0.088	0.024	4.134	0.000	0.051	0.144
SC <- D6	0.318	0.005	15.045	0.000	0.067	0.086
SC <- D7	-0.082	0.018	3.916	0.000	-0.107	-0.036
SC <- D8	0.073	0.014	3.419	0.001	0.020	0.075
R	0.127					
R2	0.124					
F-Value	37.152					
Sig						
CI	97.5%

**Table 6:** Correlation.

	Variables	1	2	3	4	5	6	7
1	Trust	1.000						
2	Recognition	0.368⁎⁎	1.000					
3	Participation	0.256⁎⁎	0.598⁎⁎	1.000				
4	Reciprocity	0.355⁎⁎	0.443⁎⁎	0.451⁎⁎	1.000			
5	Insertion	0.392⁎⁎	0.372⁎⁎	0.359⁎⁎	0.400⁎⁎	1.000		
6	Demographic	0.170⁎⁎	0.184⁎⁎					
	0.202⁎⁎	0.088⁎⁎	0.115⁎⁎	1.000				

⁎⁎ Correlation is significant at the 0.01 level (2-tailed).

## Ethics Statements

The present work was carried out in accordance with Declaration of Helsinki and informed consent was obtained from all participants. The study was approved by Institutional Review Board of Nanyang Technological University (IRB-2019-08-018-01).

## CRediT Author Statement

**Sulfikar Amir:** Conceptualization, Methodology, Original draft preparation & reviewing; **Irma Hidayana:** Methodology, Writing – draft preparation and reviewing; **Zahira Rahvenia:** Data curation, Validation; **Muhammad Salman:** Data collection and curation.

## Declaration of Competing Interest

The authors declare that they have no known competing financial interests or personal relationships that could have appeared to influence the work reported in this paper.

## Data Availability

DATASET Social Cohesion Jakarta (Original data) (DR-NTU).

## Appendix Supplementary materials

Image, application 1
